# Optimizing topical antibiotic delivery in dogs: Tear film pharmacokinetics of moxifloxacin following pre-treatment with hyaluronic acid

**DOI:** 10.1371/journal.pone.0346333

**Published:** 2026-04-10

**Authors:** Lionel Sebbag, Bar Fruchter, Daphne Kenin, Dikla Arad, Oren Pe’er

**Affiliations:** Koret School of Veterinary Medicine, The Hebrew University of Jerusalem, Rehovot, Israel; Laurentian University, CANADA

## Abstract

**Purpose:**

To evaluate the impact of pre-treatment with mucoadhesive polymers on tear film concentrations of topically administered moxifloxacin in dogs.

**Methods:**

Eight healthy dogs were enrolled in a randomized paired-eye crossover study consisting of two sessions (7-day washout). Eyes received topical 0.5% moxifloxacin alone (control) or following pre-treatment with a mucoadhesive polymer applied 30 seconds earlier: either 0.2% hyaluronic acid (HA; Hylogel^®^) or 0.75% cross-linked hyaluronic acid (XHA; Oculenis^®^). Tear samples were collected using microcapillary tubes at baseline and predefined intervals up to 480 minutes, and moxifloxacin concentrations were quantified by UV-Vis spectrophotometry (293 nm).

**Results:**

Total moxifloxacin exposure (AUC_0–480_) increased following pre-treatment with either HA formulation. The XHA produced a statistically significant 2.0-fold increase in mean AUC_0–480_ over control (47,929 vs. 24,417 µg·min/mL; P = 0.005). The HA group achieved a 1.8-fold increase in mean AUC_0–480_ (37,104 vs. 20,693 µg·min/mL), but this did not reach statistical significance (P = 0.154). In the XHA trial, a significant treatment effect (P = 0.012) and interaction (P = 0.002) were observed for concentration, which remained significantly higher than control for 15 minutes (P ≤ 0.018). In the HA trial, a significant treatment effect was noted for concentration (P = 0.020), with levels remaining significantly higher than control for 10 minutes (P ≤ 0.024). No ocular irritation was observed.

**Conclusion:**

Prior administration of mucoadhesive polymers increases moxifloxacin bioavailability on the canine ocular surface, with this effect being more pronounced for cross-linked HA (“reservoir effect”) compared to linear HA formulations. These findings may help optimize topical antibiotic delivery strategies in dogs with bacterial keratitis.

## Introduction

Topical ophthalmic solutions (“eye drops”) represent the most common route of administration for the treatment of anterior segment diseases; however, their clinical efficacy is limited by poor ocular bioavailability [1–3]. Following instillation, physiological mechanisms such as reflex tearing, blinking, and nasolacrimal drainage rapidly eliminate a large proportion of the administered drug from the ocular surface [3–5]. In dogs, tear film drug concentrations can decrease by approximately 20–46% within 1 minute and 88–97% within 30 minutes of administration [2,3,6]. Although increasing drug concentration or dosing frequency can be employed to compensate for this rapid clearance [7,8], these strategies can be challenging to implement and may increase the risk of local or systemic adverse effects. As such, alternative approaches aimed at prolonging precorneal residence time have gained increasing interest, particularly by using mucoadhesive polymers to enhance drug retention and reduce nasolacrimal drainage [9–12].

Among mucoadhesive polymers, hyaluronic acid (HA) is widely used in ophthalmology due to its excellent biocompatibility, viscoelastic properties, and capacity to interact with the ocular surface mucin layer [13–15]. However, native or linear HA exhibits a relatively short residence time on the ocular surface because of rapid clearance and enzymatic degradation [6,16,17]. To address these limitations, chemically modified formulations such as cross-linked hyaluronic acid (XHA) have been developed. XHA forms a covalently bonded three-dimensional hydrogel network with distinct rheological properties, including increased resistance to degradation and shear-thinning behavior, compared with linear HA solutions [13,17,18]. A recent fluorophotometric study in healthy dogs has demonstrated that XHA exhibits significantly prolonged ocular surface retention compared with both linear HA and saline solutions [17].

Hyaluronic acid has been shown to enhance the bioavailability of topically administered drugs through two distinct strategies: compounding and pre-treatment. Compounding, which involves direct incorporation of active drugs into HA-based vehicles, has proven effective in increasing ocular drug exposure. For example, gentamicin compounded with HA resulted in increased tear concentrations in humans [19], while compounding cefazolin or chloramphenicol with XHA significantly increased tear film concentrations and overall drug exposure in dogs [20]. However, pharmaceutical compounding is not always feasible or readily available in clinical practice. In such cases, a pre-treatment strategy using mucoadhesive polymers may represent a more convenient alternative. A recent study has shown that pre-treatment with HA or hydroxyethylcellulose prior to tropicamide instillation significantly prolongs and potentiates its mydriatic effect in dogs [21]. Despite these encouraging findings, it remains unknown whether this simple pre-treatment approach can be effectively applied to optimize topical antibiotic therapy in canine patients, a question of particular clinical relevance given the prevalence and potentially severe consequences of bacterial keratitis.

In the present study, moxifloxacin was selected as a model antibiotic due to its broad-spectrum activity against Gram-positive and Gram-negative bacteria, favorable corneal penetration, and widespread use in the management of bacterial keratitis in both human and veterinary medicine [22,23]. In addition, moxifloxacin is well suited for use with HA–based delivery systems, as prior ocular studies have demonstrated stable incorporation and sustained release of moxifloxacin from HA matrices, including intraocular implant models [24]. Collectively, these characteristics make moxifloxacin a clinically relevant candidate in which improved ocular surface exposure could translate into meaningful therapeutic benefit. Accordingly, the objective of this study was to compare tear film exposure of 0.5% moxifloxacin administered alone versus following pre-treatment with either linear HA or cross-linked HA. We hypothesized that HA pre-treatment would enhance moxifloxacin bioavailability, with XHA producing the greatest effect due to its superior ocular surface retention properties.

## Methods

### Animals

An a priori sample size calculation was based on previously published data evaluating HA pre-treatment in dogs [21], where the mean difference in drug AUC between control and HA-pretreated eyes was 4.1 with a standard deviation of 2.3. Assuming a power of 90% and an alpha of 0.05, a paired t-test (SigmaPlot version 15.0; Systat Software Inc.) indicated that a minimum of six dogs would be sufficient to detect a statistically significant difference. A total sample size of *n* = 8 dogs was therefore selected to account for known intra- and inter-individual variability in tear film drug concentrations [2,3,20]. Eight healthy Labrador Retrievers (2 males, 6 females; 16 eyes) were recruited from the Israeli Guide Dog Center for the Blind. Dogs were aged 1.6 ± 0.2 years and weighed 29.4 ± 4.0 kg. Prior to study inclusion, all dogs were confirmed to be healthy based on a complete physical and ophthalmic examination performed by a board-certified veterinary ophthalmologist (LS), including slit-lamp biomicroscopy (SL-17; Kowa Company), rebound tonometry (TonoVet Plus; Icare Finland), and indirect ophthalmoscopy (Keeler Vantage; Keeler Instruments). Dogs were excluded if they had any evidence of ocular disease, known drug sensitivities, or systemic illness. The dogs were group-housed in a climate-controlled environment with a 12-hour light/dark cycle. No animals were sacrificed in this study. All procedures were performed in conscious dogs using gentle manual restraint only, without sedation, anesthesia, or analgesia, as tear sampling with microcapillary tubes is minimally invasive and non-painful. Dogs were handled by experienced personnel and provided with breaks as needed to minimize stress. The study was approved by the Institutional Animal Care and Use Committee of Israel’s Ministry of Health (OPRR-A01-5011), and signed consent was obtained from the owners.

### Experimental design

The study utilized a randomized, crossover design consisting of two experimental sessions separated by a 7-day washout period to ensure complete drug clearance and avoid potential carryover effects from the HA-antibiotic interaction. In the first session, one eye of each dog was randomly selected to receive a single drop of 0.2% linear sodium hyaluronate (Hylogel^®^; Kivema Ltd., Kadima, Israel). Thirty seconds later, one drop of 0.5% moxifloxacin (Vigamox^®^, Alcon Inc., Fort Worth, TX, USA) was administered to both eyes. In the second session, the same eye was assigned to receive a single drop of 0.75% cross-linked hyaluronic acid (Oculenis^®^; Dômes Pharma Inc., France). Thirty seconds thereafter, one drop of 0.5% moxifloxacin was instilled into both eyes. The same eye that received HA in the first session also received XHA in the subsequent session, allowing within-eye comparison of the two polymers.

### Tear collection and drug quantification

Tear fluid was collected from both eyes at baseline (t = 0 min; immediately following antibiotic instillation and a spontaneous blink), and subsequently at 1, 5, 10, 15, 30, 60, 120, 240, 360, and 480 min post-instillation. A 2-μL microcapillary glass tube (Drummond Scientific Co., Broomall, PA, USA) was placed in contact with the inferior lacrimal lake for ≤ 5 seconds to collect tear fluid by capillary action, with care taken to minimize reflex tearing. Moxifloxacin concentrations were quantified using UV-Vis spectrophotometry (NanoDrop One; Thermo Fisher Scientific Inc., Waltham, MA, USA). Standard calibration curves were generated in blank tear fluid using serial dilutions of a moxifloxacin reference standard (Moxifloxacin USP; Sigma-Aldrich, St. Louis, MO, USA) over a concentration range of 0.1–10,000 μg/mL. Blank tears were collected from the same dogs prior to the experiment using ophthalmic sponges, as previously described [2]. Absorbance was measured at a wavelength of 293 nm, in accordance with previously validated methods for moxifloxacin quantification [25–27]. Further, to evaluate potential matrix effects related to the presence of HA and XHA, additional validation experiments were performed. UV-Vis spectra were recorded from 220 to 400 nm for blank canine tears, HA, XHA, and tear-polymer mixtures to assess potential spectral overlap at the analytical wavelength. Analytical accuracy and precision were further evaluated using spike-recovery experiments performed at 10, 100, and 500 μg/mL in each matrix, with all measurements obtained in triplicate. Mean recovery and coefficient of variation (CV) were calculated across spike levels. Limits of detection (LOD) and limits of quantification (LOQ) were estimated from the low-concentration range of the calibration data.

### Data analysis

Data normality was confirmed using the Shapiro–Wilk test (P ≥ 0.096), and results are presented as mean ± standard deviation (range). For each experimental session (linear hyaluronic acid or cross-linked hyaluronic acid), moxifloxacin tear concentrations were analyzed using two-way repeated-measures ANOVA with Treatment (HA-pretreated vs contralateral control) and Time as within-subject factors, followed by Bonferroni post hoc testing. Total tear exposure over 0–480 minutes (AUC₀-₄₈₀) was calculated using the trapezoidal method and compared between eyes using paired t-tests. Statistical analyses were performed with SigmaPlot version 15.0 (Systat Software Inc.), with significance set at P < 0.05.

## Results

### UV-Vis spectrophotometry

UV-Vis spectral scans of blank tears, HA, XHA, and tear-polymer mixtures showed no distinct absorbance peaks and minimal baseline absorbance (≤ 0.02 AU) in the region surrounding the analytical wavelength (290–295 nm), indicating no relevant spectral interference with moxifloxacin detection at 293 nm. Analytical accuracy was confirmed by spike-recovery experiments, with mean recoveries ranging from 97.7% to 99.1% across all matrices and coefficients of variation ≤ 2.2%, confirming good analytical precision. The calibration curve exhibited high linearity (R^2^ = 0.995), the LOD was 3.3 µg/mL and the LOQ was 10.0 µg/mL.

### Linear HA

**Fig 1:**
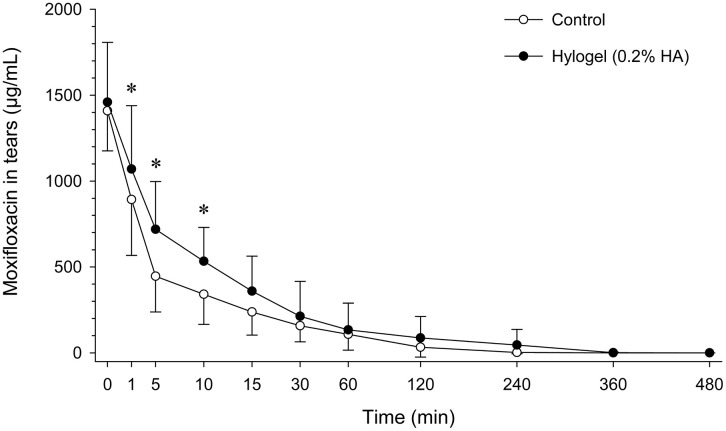
Tear film concentrations of moxifloxacin in canine eyes following a single topical instillation of 0.5% moxifloxacin administered alone (control; white circles) or after pre-treatment with 0.2% linear hyaluronic acid (Hylogel^®^; black circles) applied 30 seconds earlier. Data are presented as mean ± SD. Asterisks indicate time points at which moxifloxacin concentrations were significantly higher in HA-pretreated eyes compared with control eyes (P < 0.05).

**Fig 2:**
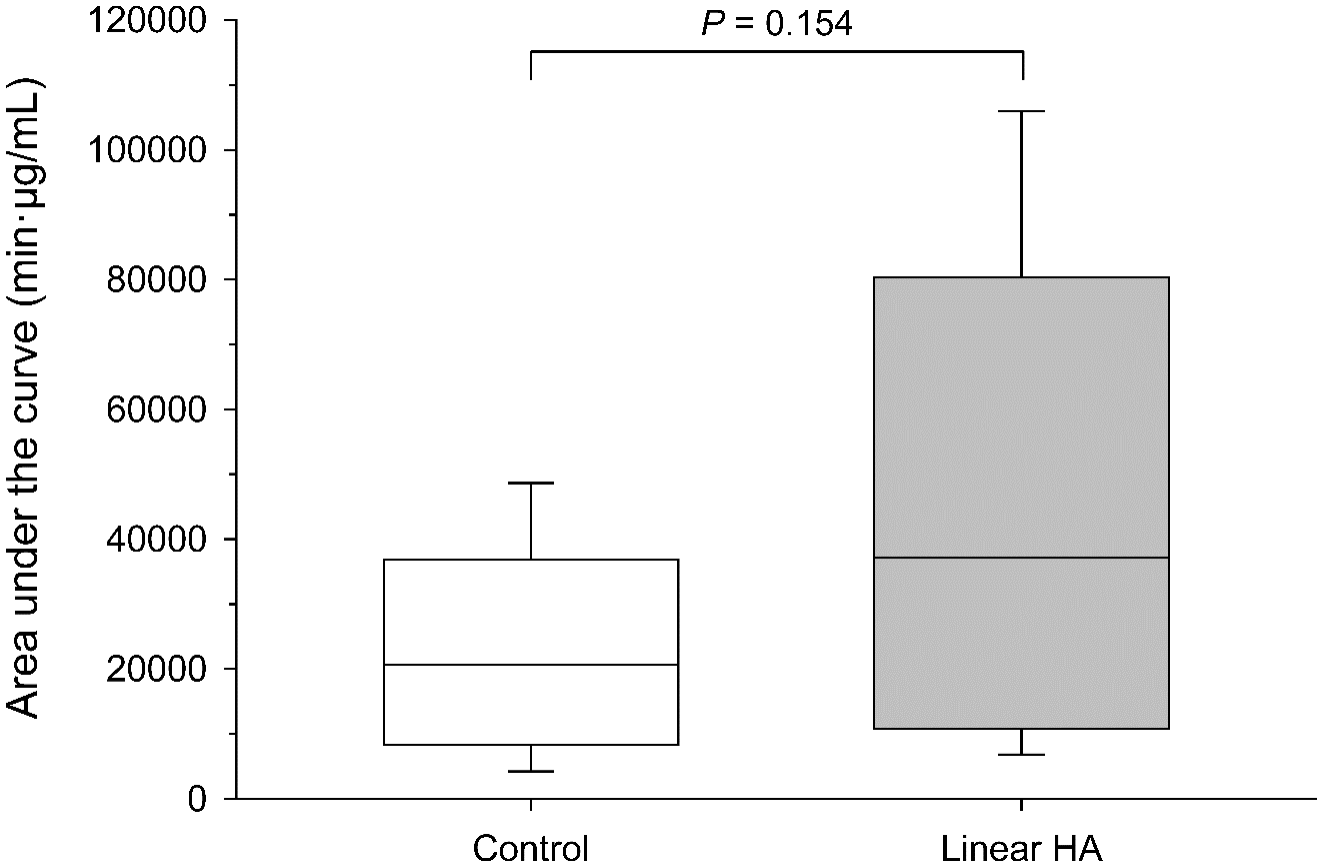
Box-and-whisker plot depicting the area under the concentration-time curve (AUC_0-480_) in tear film following a single topical instillation of 0.5% moxifloxacin administered alone (control; white box) or after pre-treatment with 0.2% linear hyaluronic acid (Hylogel^®^; gray box) applied 30 seconds earlier. Each plot represents the mean (horizontal line), minimum (lower whisker), 25th percentile (lower limit of box), 75th percentile (upper limit of box), and maximum (upper whisker).

Mean ± SD concentrations of moxifloxacin in tears were significantly higher in eyes pre-treated with linear HA compared to control eyes at 1 min (1070 ± 369 μg/mL vs. 893 ± 325 μg/mL; *P* = 0.024), 5 min (719 ± 279 μg/mL vs. 446 ± 208 μg/mL; *P* < 0.001), and 10 min (533 ± 196 μg/mL vs. 341 ± 176 μg/mL; *P* = 0.015) post-instillation (Fig 1). No differences were noted between groups at baseline (t = 0 min) and from 15 min onward (*P* ≥ 0.119). The AUC₀-₄₈₀ was approximately 1.8-fold higher in eyes pre-treated with linear HA (37,104 ± 34,774 µg·min/mL) compared with control (20,693 ± 13,891 µg·min/mL), although this difference was not statistically significant (*P* = 0.154; Fig 2).

### Cross-linked HA

**Fig 3:**
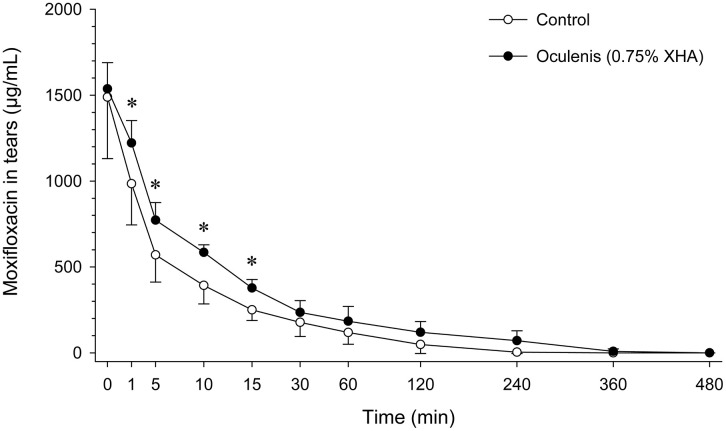
Tear film concentrations of moxifloxacin in canine eyes following a single topical instillation of 0.5% moxifloxacin administered alone (control; white circles) or after pre-treatment with 0.75% cross-linked hyaluronic acid (Oculenis^®^; black circles) applied 30 seconds earlier. Data are presented as mean ± SD. Asterisks indicate time points at which moxifloxacin concentrations were significantly higher in XHA-pretreated eyes compared with control eyes (P < 0.05).

**Fig 4:**
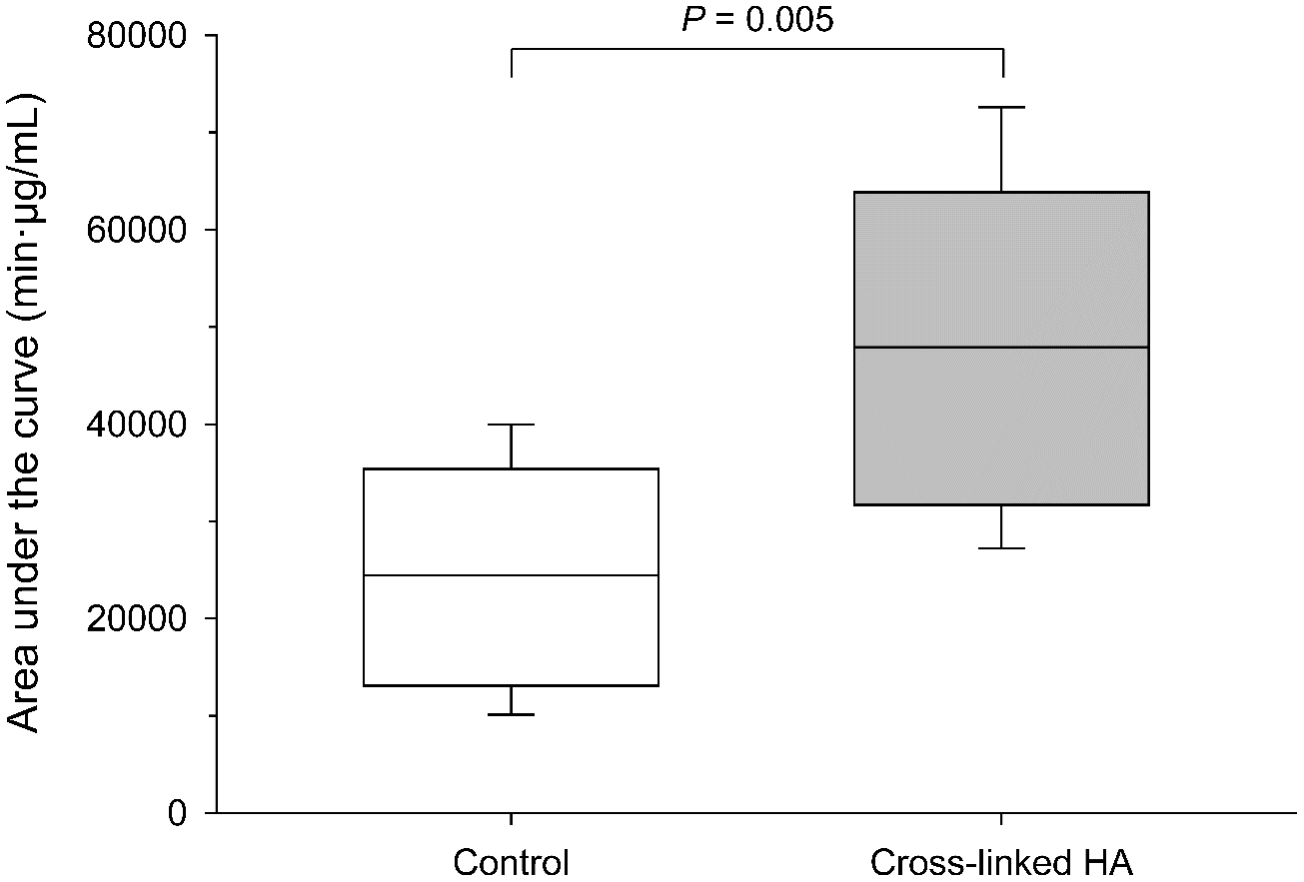
Box-and-whisker plot depicting the area under the concentration-time curve (AUC_0-480_) in tear film following a single topical instillation of 0.5% moxifloxacin administered alone (control; white box) or after pre-treatment with 0.75% cross-linked hyaluronic acid (Oculenis^®^; gray box) applied 30 seconds earlier. Each plot represents the mean (horizontal line), minimum (lower whisker), 25th percentile (lower limit of box), 75th percentile (upper limit of box), and maximum (upper whisker).

Mean ± SD concentrations of moxifloxacin in tears were significantly higher in eyes pre-treated with XHA compared to control eyes at 1 min (1222 ± 131 vs. 985 ± 240 μg/mL; *P* < 0.001), 5 min (772 ± 103 vs. 570 ± 158 μg/mL; *P* < 0.001), 10 min (585 ± 44 vs. 393 ± 108 μg/mL; *P* < 0.001), and 15 min (377 ± 50 vs. 250 ± 62 μg/mL; *P* = 0.018) post-instillation (Fig 3). No differences were noted between groups at baseline (t = 0 min) and from 30 min onward (*P* ≥ 0.177). The AUC₀-₄₈₀ was approximately 2.0-fold higher in eyes pre-treated with XHA (47,929 ± 15,451 µg·min/mL) compared to control (24,417 ± 10,298 µg·min/mL), a difference that was statistically significant (*P* = 0.005; Fig 4).

### Linear HA vs. Cross-linked HA

When directly comparing eyes pre-treated with linear HA versus XHA, mean tear concentrations of moxifloxacin were generally higher in XHA-pretreated eyes, although no statistically significant differences were detected between formulations at any individual time point (*P* ≥ 0.105). Overall AUC₀-₄₈₀ was approximately 1.3-fold higher following pre-treatment with XHA compared to linear HA, but this difference did not reach statistical significance (*P* = 0.416).

## Discussion

This randomized, paired-eye crossover study demonstrates that a simple sequential pre-treatment strategy with mucoadhesive polymers can enhance the tear film exposure of topically administered 0.5% moxifloxacin in dogs. Pre-treatment with 0.75% cross-linked hyaluronic acid produced a statistically significant ~2.0-fold increase in AUC₀-₄₈₀ compared with control eyes and maintained higher tear concentrations for the first 15 minutes after dosing. Pre-treatment with 0.2% linear HA yielded a comparable effect (mean ~1.8-fold AUC increase) and higher early concentrations (up to 10 minutes), but the AUC increase did not reach statistical significance. When comparing XHA versus linear HA directly, tear concentrations were generally higher with XHA and AUC was ~ 1.3-fold higher, yet differences were not statistically significant at any individual time point. Collectively, these data support the concept that “priming” the ocular surface with a mucoadhesive matrix can create a short-lived but clinically meaningful reservoir effect [17,21], with the most consistent benefit observed for XHA.

Topical solutions remain the mainstay for anterior segment therapy, but ocular bioavailability is intrinsically low because drug is rapidly removed by blinking, reflex tearing, and nasolacrimal drainage. In dogs, fluorophotometric and pharmacokinetic studies consistently demonstrate rapid clearance of topically instilled eye drops, with tear film drug levels decreasing by approximately 20–46% within the first minute and by 88–97% within 30 minutes after administration [2,3,6]. The current study reproduces this fundamental kinetic pattern for moxifloxacin and highlights why strategies targeting the first 10–15 minutes post-instillation can disproportionately influence overall exposure. In that context, the observation that HA pre-treatment increased concentrations during this early clearance window is biologically coherent and pharmacologically relevant.

Mucoadhesive polymers enhance drug delivery primarily by prolonging precorneal residence time through noncovalent interactions (*e.g.*, hydrogen bonding, electrostatic interactions) and chain entanglement with the mucus layer, thereby delaying clearance and increasing time for absorption or tissue partitioning [28,29]. Hyaluronic acid is particularly attractive because it is a naturally occurring glycosaminoglycan with excellent ocular tolerability and demonstrated benefits as a lubricant and epithelial-supportive biopolymer [14]. Importantly, the strategy used here is pre-treatment, not compounding. Thus, the effect depends on how much polymer remains on the ocular surface at the moment the antibiotic drop arrives, and whether it can transiently retain the antibiotic before it is drained. Notably, baseline moxifloxacin concentrations did not differ between control and polymer-pretreated eyes, indicating that the initial HA layer did not dilute the subsequently administered antibiotic.

Cross-linking transforms HA into a three-dimensional hydrogel network with altered rheological properties (including shear-thinning behavior), increased resistance to enzymatic degradation, and prolonged ocular surface contact time compared with linear HA [14,18]. In healthy dogs, fluorophotometric work has shown that XHA persists longer on the ocular surface than linear HA or saline, with signal detectable up to 180 minutes and a distinctive distribution pattern evolving from broad coverage to accumulation in the tear meniscus and medial canthus [17]. These characteristics provide a plausible mechanistic basis for our finding that XHA maintained higher moxifloxacin concentrations for a longer early interval (15 minutes vs 10 minutes for linear HA) and achieved a significant AUC gain. However, the magnitude of difference between XHA and linear HA in this sequential model remained modest, and direct comparisons were not statistically significant. This finding is not surprising. Unlike “drug-in-vehicle” approaches, pre-treatment creates only a transient interface between polymer and antibiotic. Once the moxifloxacin drop is instilled, both polymer layers are rapidly diluted by the added volume and tear turnover. Under these constraints, detecting clear separation between two mucoadhesive systems may require larger sample sizes, different dosing intervals, or disease models where tear film dynamics are altered [21]. An additional consideration is that the two HA formulations differed not only in polymer architecture but also in concentration (0.2% vs 0.75%). Higher polymer mass and viscosity may independently influence precorneal residence time. Because the study compared clinically available products, the present design does not fully separate cross-linking effects from concentration-dependent influences.

The current data should be interpreted alongside studies using HA as the vehicle for antibiotics. In humans, gentamicin delivered in a sodium hyaluronate vehicle increased early tear concentrations compared with buffer [19]. In dogs, compounding cefazolin and chloramphenicol into XHA produced substantially higher tear concentrations and significantly higher AUC compared with a conventional polymer vehicle [20]. Those results support a key principle: the greatest gains occur when the drug remains physically associated with the mucoadhesive matrix from the outset. Pre-treatment, by contrast, is simpler and broadly feasible, but inherently constrained by sequential dilution and drainage. Still, the present findings indicate that pre-treatment can deliver measurable benefits, especially with XHA, and may represent a practical “middle ground” when compounding is unavailable or impractical.

Moxifloxacin is a fourth-generation fluoroquinolone widely used for ocular infections due to broad Gram-positive and Gram-negative activity and favorable clinical performance as a topical agent [22,23]. Because topical moxifloxacin already achieves high early tear concentrations, a ceiling effect is plausible in healthy eyes: once concentrations are well above bacterial MICs, increasing exposure may not translate into dramatic additional benefit in normal ocular surface conditions. That said, bacterial keratitis is a different biological environment. Corneal epithelial disruption and inflammation can change permeability and tear film dynamics, and higher early retention may improve stromal delivery during the period when drug is most rapidly cleared. Moreover, optimizing exposure without increasing dose or frequency is attractive from both a compliance and stewardship standpoint, particularly as clinicians aim to preserve fluoroquinolone utility [23].

A major strength of the present approach is clinical usability. A 30-second interval between polymer and antibiotic was intentionally selected as a realistic “home-care” workflow. We selected a 30-second interval as a compromise between real-world practicality and ocular pharmacokinetics: it approximates the time an owner realistically needs to switch bottles, remains well within the first minute during which ~20–46% of a drop is already cleared from the ocular surface [2,3,6], and builds on prior work showing enhanced drug exposure with HA pre-treatment as early as 10 seconds [21]; however, that 10-second interval was achieved under controlled experimental conditions with dedicated assistance and is unlikely to be reproducible in routine home administration. From a practical standpoint, owners can readily apply an HA-based lubricant drop and then administer antibiotic shortly after, without complex compounding or additional devices. The finding that early tear concentrations and overall exposure increase with this workflow, with no observed ocular irritation, supports feasibility. While the statistically significant AUC gain occurred with XHA, the linear HA results still suggest potential benefit, especially given that the study was conducted in healthy eyes with intact clearance mechanisms and that inter-individual variability in tear sampling can be substantial.

Several limitations should be noted. First, the study was performed in ophthalmologically healthy dogs, which limits generalizability to clinical cases where tearing, ocular discomfort, and surface irregularity may alter both polymer residence time and sampling variability. Second, only one pre-treatment interval (30 seconds) and single-dose administration were evaluated; the “effective window” for sequential dosing may be shorter or longer than 30 seconds, depending on polymer retention and tear film dynamics, and delineating this window would be clinically valuable. Third, only one concentration and brand/formulation of each HA type was tested; rheology and performance vary across commercial products [14,18]. Finally, outcomes were based on tear film pharmacokinetics; corneal tissue concentrations and clinical endpoints (e.g., healing time, microbial clearance) were not assessed and remain important next steps.

Future studies should evaluate *(i)* diseased eyes, particularly clinical patients with bacterial keratitis; *(ii)* additional pre-treatment intervals and repeated dosing; and *(iii)* comparisons between pre-treatment and compounded formulations, including whether XHA pre-treatment can reduce the required dosing frequency while maintaining therapeutic exposure. Because XHA has shown prolonged surface retention in dogs [17] and strong performance as an antibiotic vehicle [20], it is also worth testing whether pre-treatment offers additive benefit when combined with other delivery-optimizing strategies already used in practice.

In conclusion, pre-treatment with mucoadhesive polymers enhances tear film exposure of topical moxifloxacin in dogs, with the most consistent and statistically robust effect observed for cross-linked HA. Although XHA tended to outperform linear HA, differences between the two polymers were modest in this sequential model. Clinically, a simple HA “priming” step offers a feasible approach to optimize topical antibiotic delivery, particularly when compounding is not available, and warrants further evaluation in canine eyes with bacterial keratitis where improved retention may translate into improved outcomes.

## Supporting information

S1 Data Moxifloxacin and HA in canine tears. (XLSX)
